# Supplementary material for: Survey of nucleotide-specific Rab GTPase interactions reveals multiple Rab effectors
Source: bioRxiv. 2026 Oct 8:2026.09.15.751902. Originally published 2026 Sep 17. Preprint. [Version 2] doi: 10.64898/2026.09.15.751902 (PMC13596360; doi:10.64898/2026.09.15.751902)

bioRxiv preprint doi: https://doi.org/10.64898/2026.09.15.751902; this version posted October 8, 2026. The copyright holder for this preprint (which was not certified by peer review) is the author/funder, who has granted bioRxiv a license to display the preprint in perpetuity. It is made available under a CC-BY 4.0 International license.

Supplementary Table 1. Plasmids. Plasmids used in this study

Supplementary Table 2. Non-specific, non-relevant interactors. Compiled list of prey fragments eliminated from the list of potential interacting partners and the criteria used for their elimination.

bioRxiv preprint doi: https://doi.org/10.64898/2026.09.15.751902; this version posted October 8, 2026. The copyright holder for this preprint (which was not certified by peer review) is the author/funder, who has granted bioRxiv a license to display the preprint in perpetuity. It is made available under a CC-BY 4.0 International license.

Supplementary Table 3. Candidate Rab interactors. Interactors are shown for each Rab GTPase, designated for those that bind the GTP and/or the GDP conformation. Interactions previously described in BioGrid are in blue.

Supplementary Figure 1. Absence of self-activation by Rab GTPase baits.

A. Binary Y2H spot assays of each Rab GTPase bait (GTP-locked Q>L or GDP-locked S/T>N) paired with empty Gal4 activation domain vector, spotted onto permissive (+His) and selective (−His) plates. Absence of growth on −His confirms that no bait construct self-activates the reporter. Asterisks (*) denote Rab1 and Rab18, expressed from the reduced-strength *Tef1** promoter. Vector-only control is denoted (Ø).

B. Anti-myc immunoblots of yeast transformants expressing the indicated p*TEF1*-GBD and p*Tef1** fusion proteins. Two colonies of each transformant pool were analyzed. Only transformants verified for expression of the bait fusion were used in DEEPN runs.

Supplementary Figure 2. Full binary Y2H panel for MICAL-L1 and OPTN.
Binary Y2H spot assays for MICAL-L1 C-terminal fragment (aa 523-838, bMERB-containing) and OPTN N-terminal fragment (aa 45-339) against the complete Rab GTPase panel in GTP-locked (Q, Q>L) and GDP-locked (T, S/T>N) conformations on permissive (+His) and selective (−His) plates. Positive interactions are highlighted green. Vector-only control is denoted (Ø).

Supplementary Figure 3. Full binary Y2H panel for RABEP1.
Binary Y2H spot assays for RABEP1 fragment i (aa 62-261) and fragment ii (aa 222-393) against the complete Rab GTPase panel on permissive (+His) and selective (−His) plates, for both GTP-locked (Q>L) and GDP-locked (S/T>N) Rab baits. Fragment i shows GTP-dependent interaction with multiple Rabs; fragment ii shows selective interaction with Rab5 and weak interaction with Rab8. Positive Y2H interactions are highlighted in green. Vector-only control is denoted (Ø).

Supplementary Figure 4. Full binary Y2H Rab panel for APPL2.

Supplementary Figure 5. Full binary Y2H Rab panel for RAB11FIP2.

Supplementary Figure 6. Full binary Y2H Rab panel for RUFY2.

Supplementary Figure 7. Full binary Y2H Rab panel for IFT81.

Supplementary Figure 8. Full binary Y2H Rab panel for SNX29.

Supplementary Figure 9. Full binary Y2H Rab panel for HPS4.

Supplementary Figure 10. Full binary Y2H Rab panel for DAB2IP.

Supplementary Figure 11. Full binary Y2H Rab panel for BBS7.

Supplementary Figure 12. Full binary Y2H Rab panel for SYN3.

Supplementary Figure 13. A-C. Vector-only control is denoted (Ø). **A.** Binary screens of the Rab panel with the SNX13-RGS domain recovered from the DEEPN screen. **B.** Binary screens of the Rab panel of the GTP-bound conformation against the SNX14 RGS domain. **C.** Y2H interaction of the indicated Rab11 mutants with vector alone, the SNX14 RGS domain, or the RGS domain with the E370K mutation. Both the S20V and the Q70L mutations are thought to lock Rab11 in a GTP conformation. However, the S20V mutation on its own blocks binding of Rab11 to SNX14 RGS domain and it diminishes binding to the Q70L mutant when in combination. **D.** Levels of the wild type and E370K mutant SNX14 RGS Gal4 activation domain fusion protein bait in duplicate transformants. **E.** Levels of the SNX14 RGS domain with

bioRxiv preprint doi: https://doi.org/10.64898/2026.09.15.751902; this version posted October 8, 2026. The copyright holder for this preprint (which was not certified by peer review) is the author/funder, who has granted bioRxiv a license to display the preprint in perpetuity. It is made available under a CC-BY 4.0 International license.

phosphorylation sites mutated to Alanine or Aspartate compared to wild type SNX14 RGS. **F.** Immunoblots of the indicated Rab11 Gal4-GBD fusion proteins used in C. **G.** Pooled and dialyzed samples used for NMR analysis were analyzed by SDS-PAGE and Coomassie blue staining.

Supplemental Figure 1
Absence of self-activation by Rab GTPase 'baits'

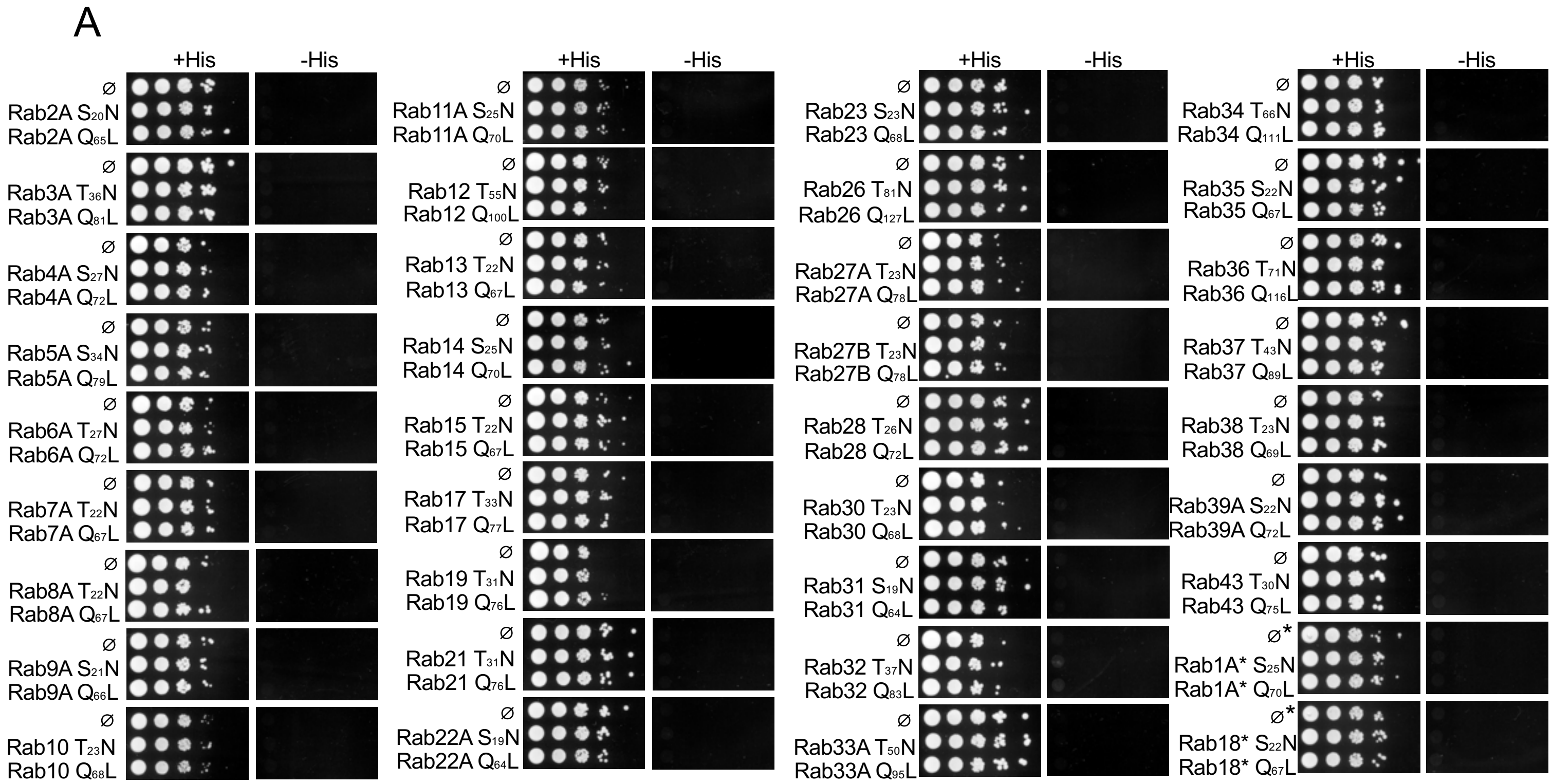

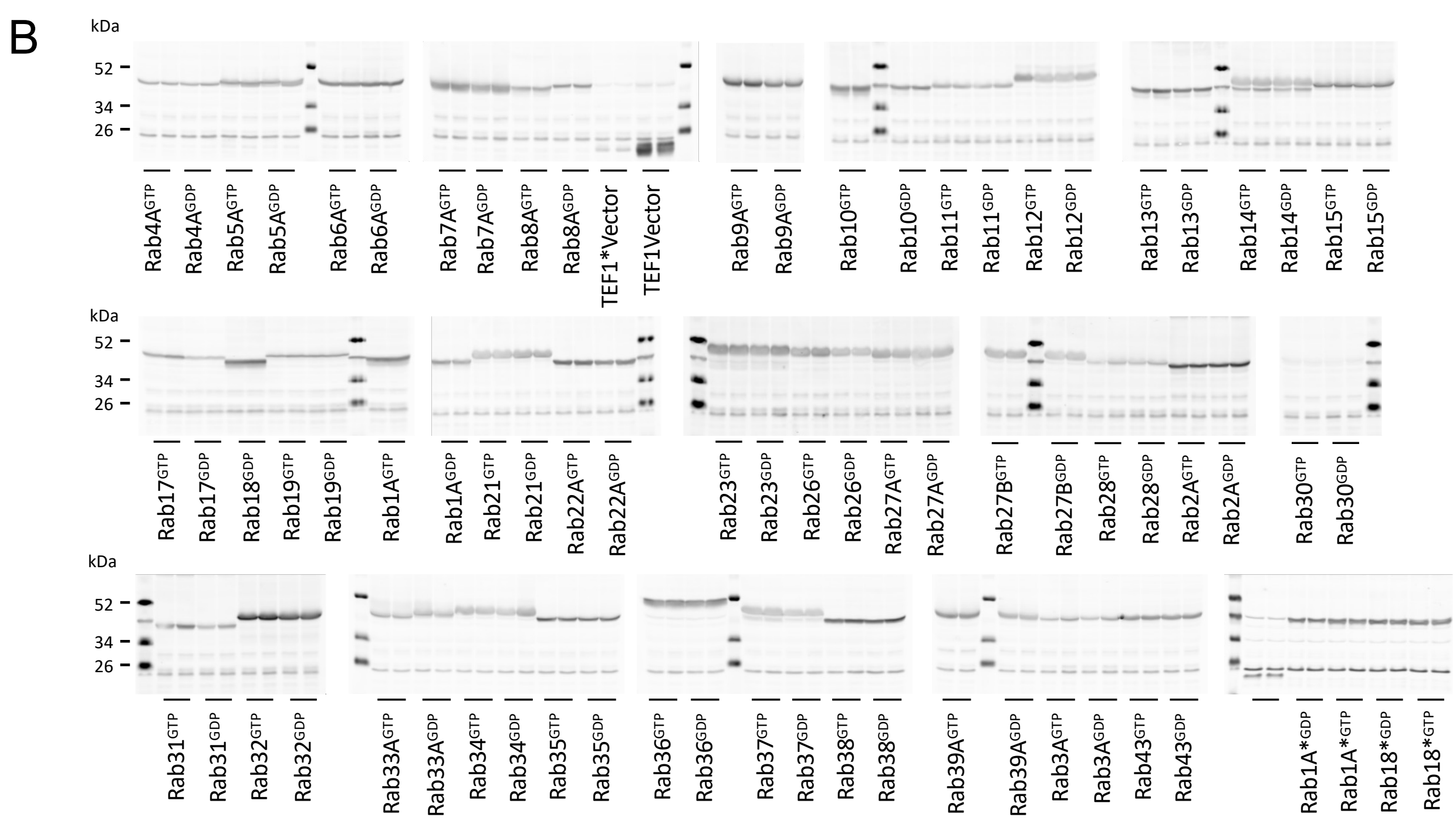

Supplemental Figure 2

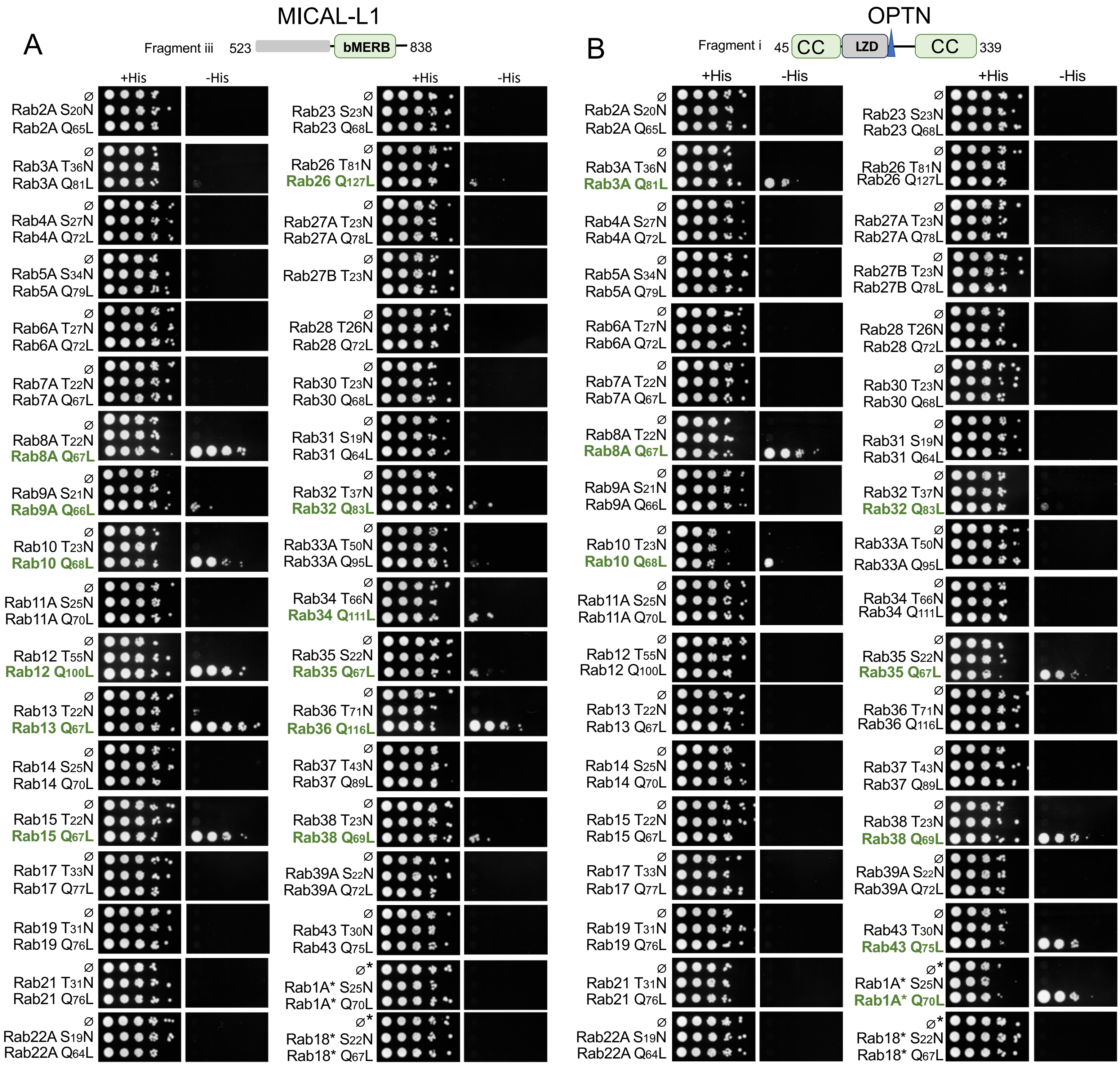

Supplemental Figure 3

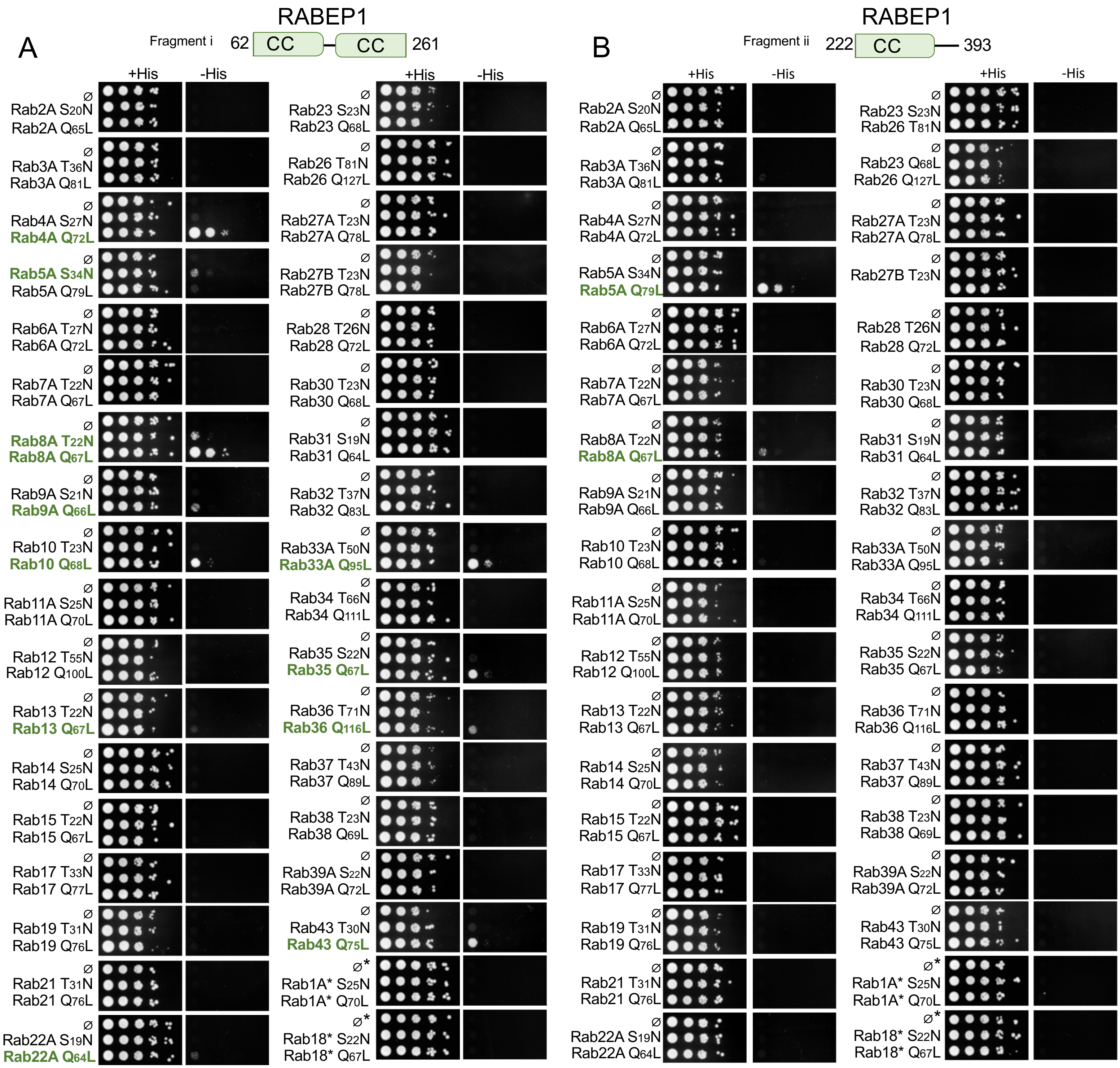

Supplemental Figure 4

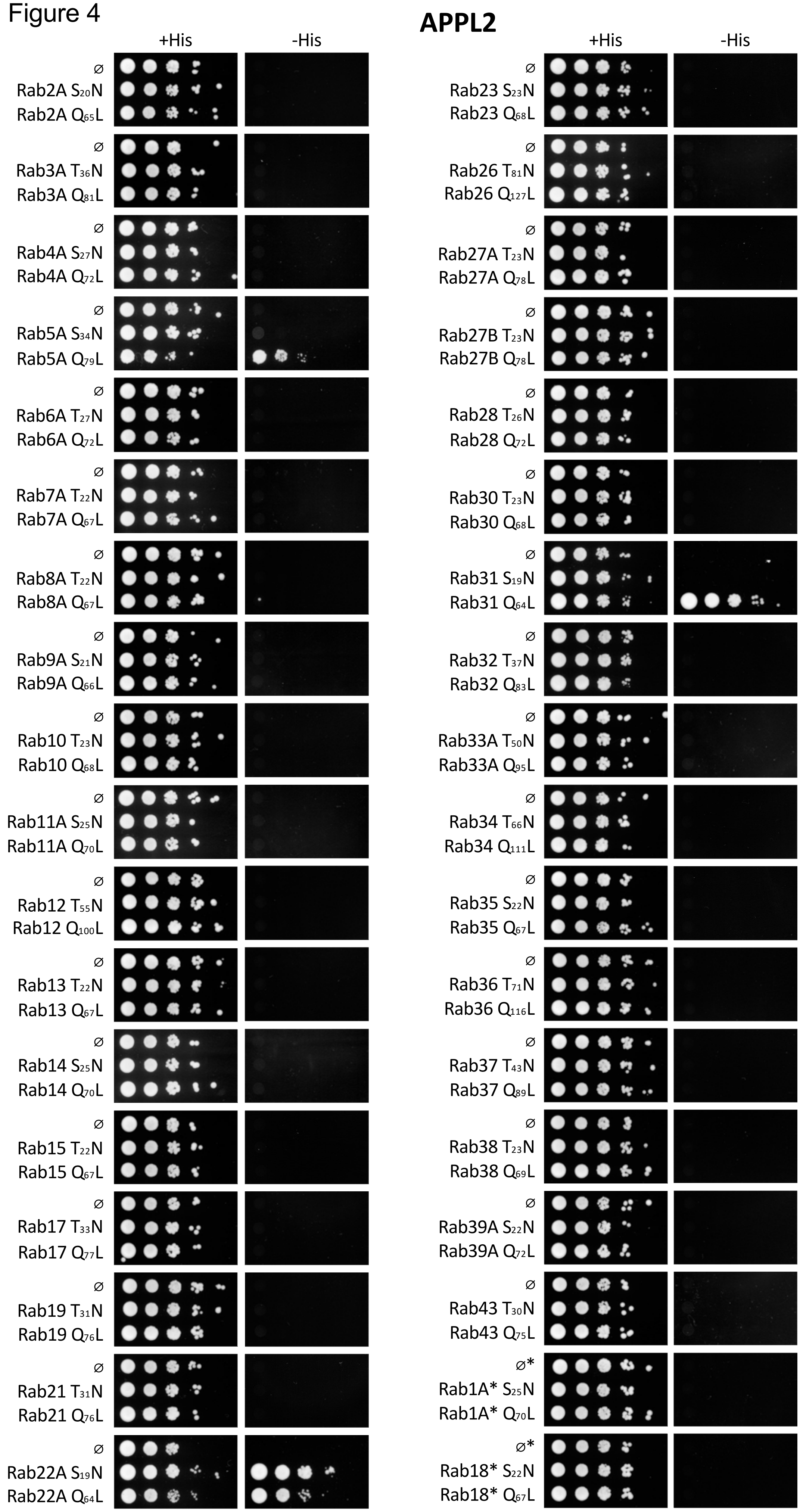

Supplemental Figure 5

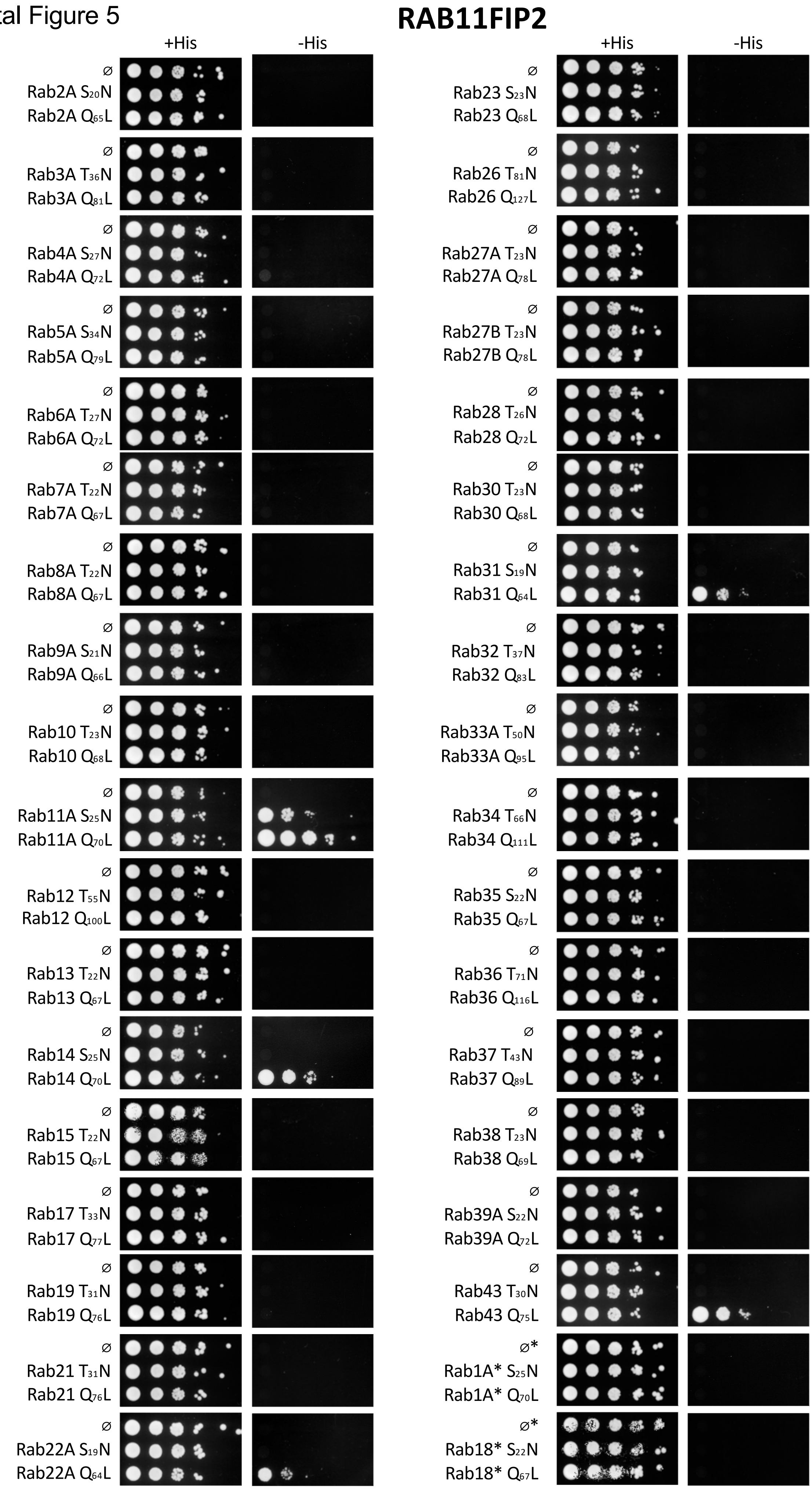

Supplemental Figure 6

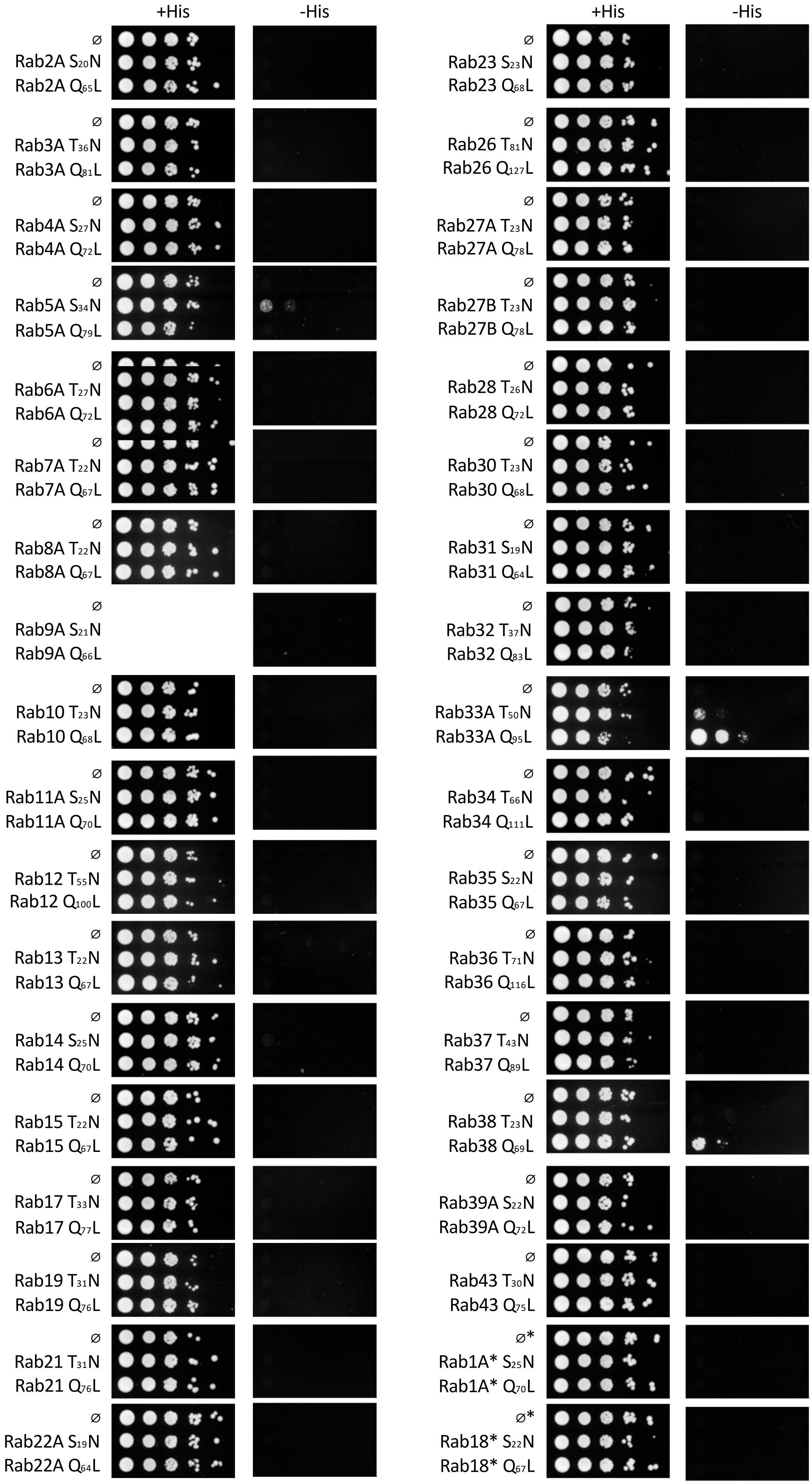

Supplemental Figure 7

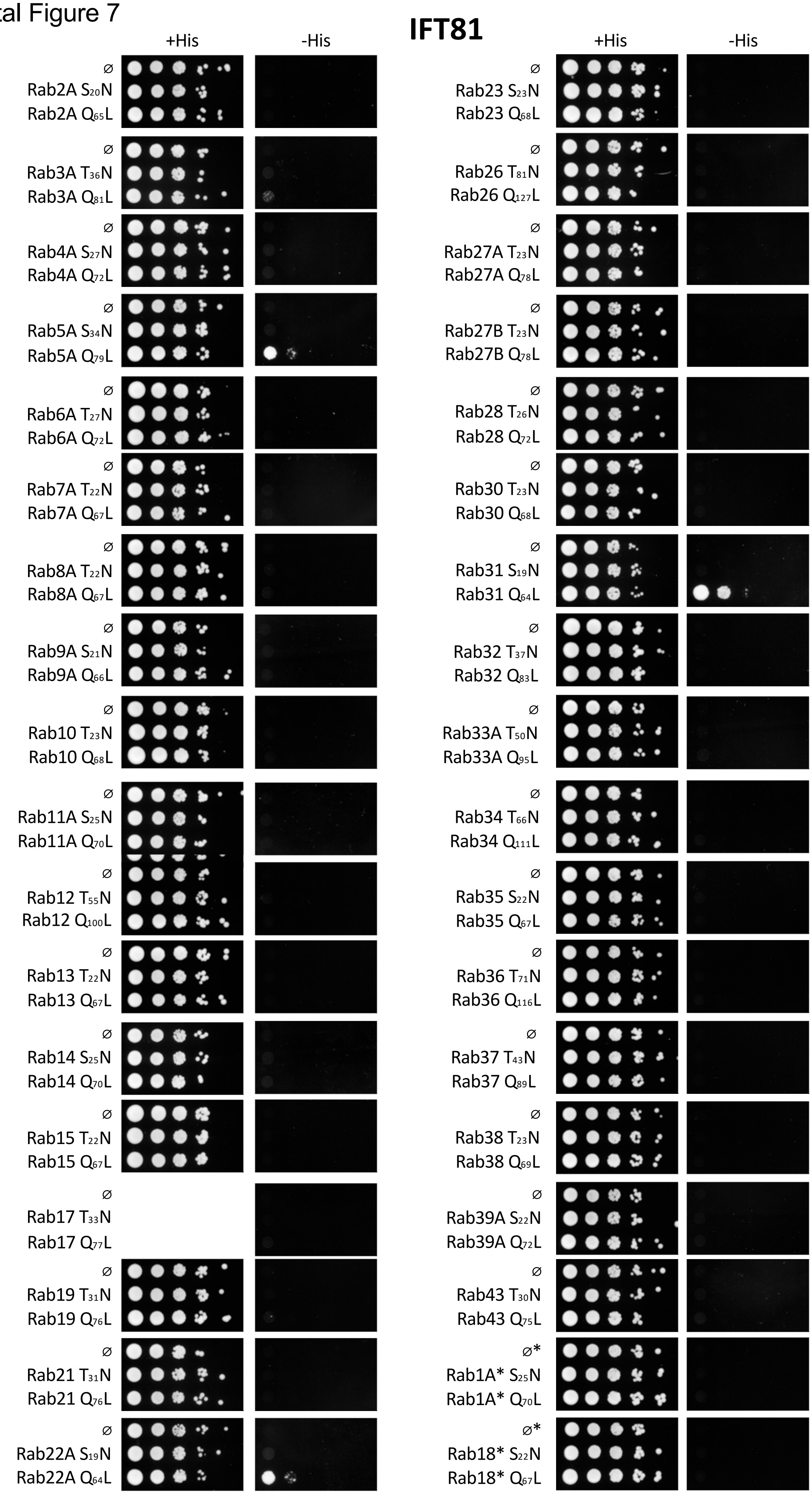

Supplemental Figure 8

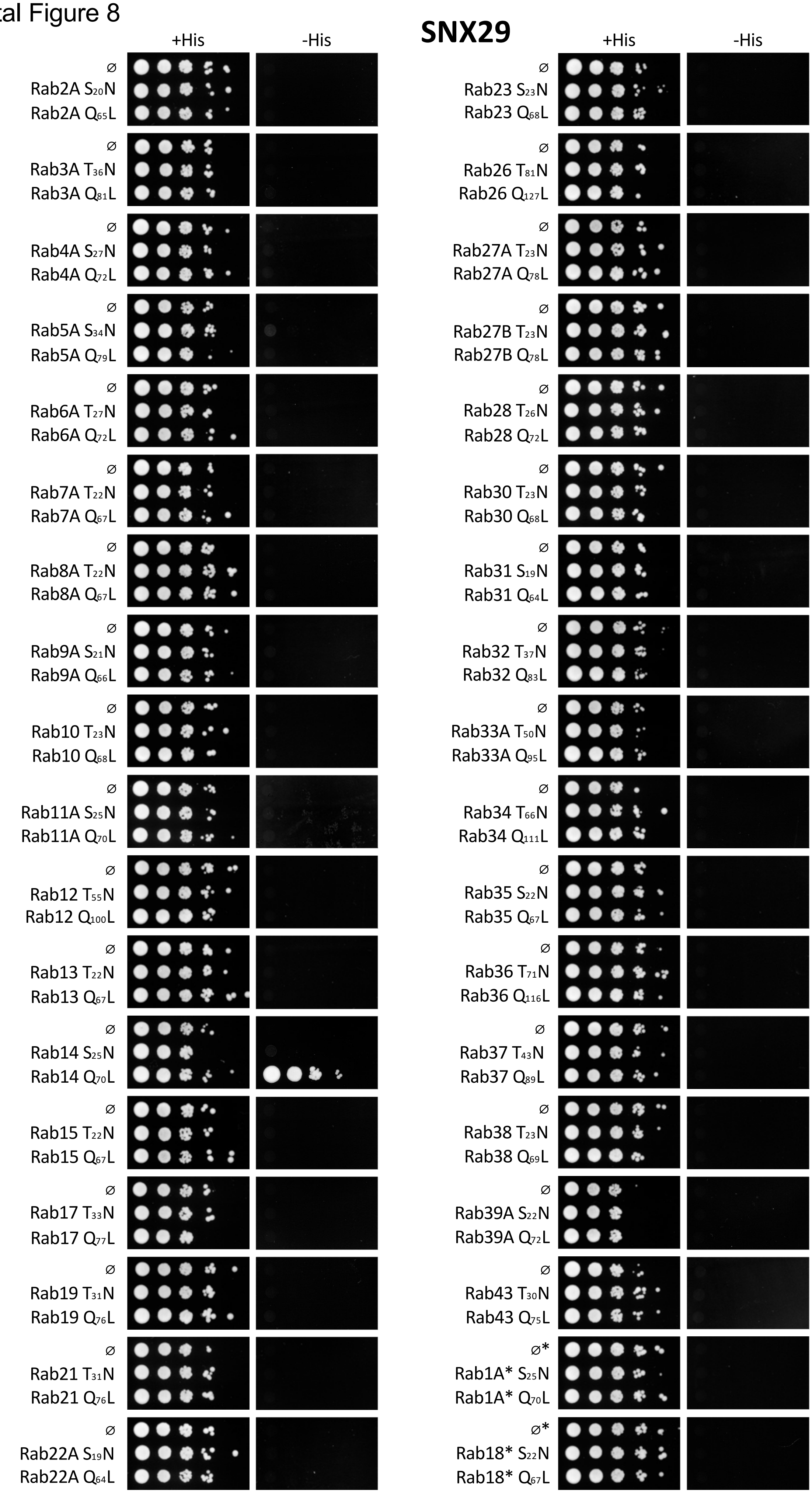

Supplemental Figure 9

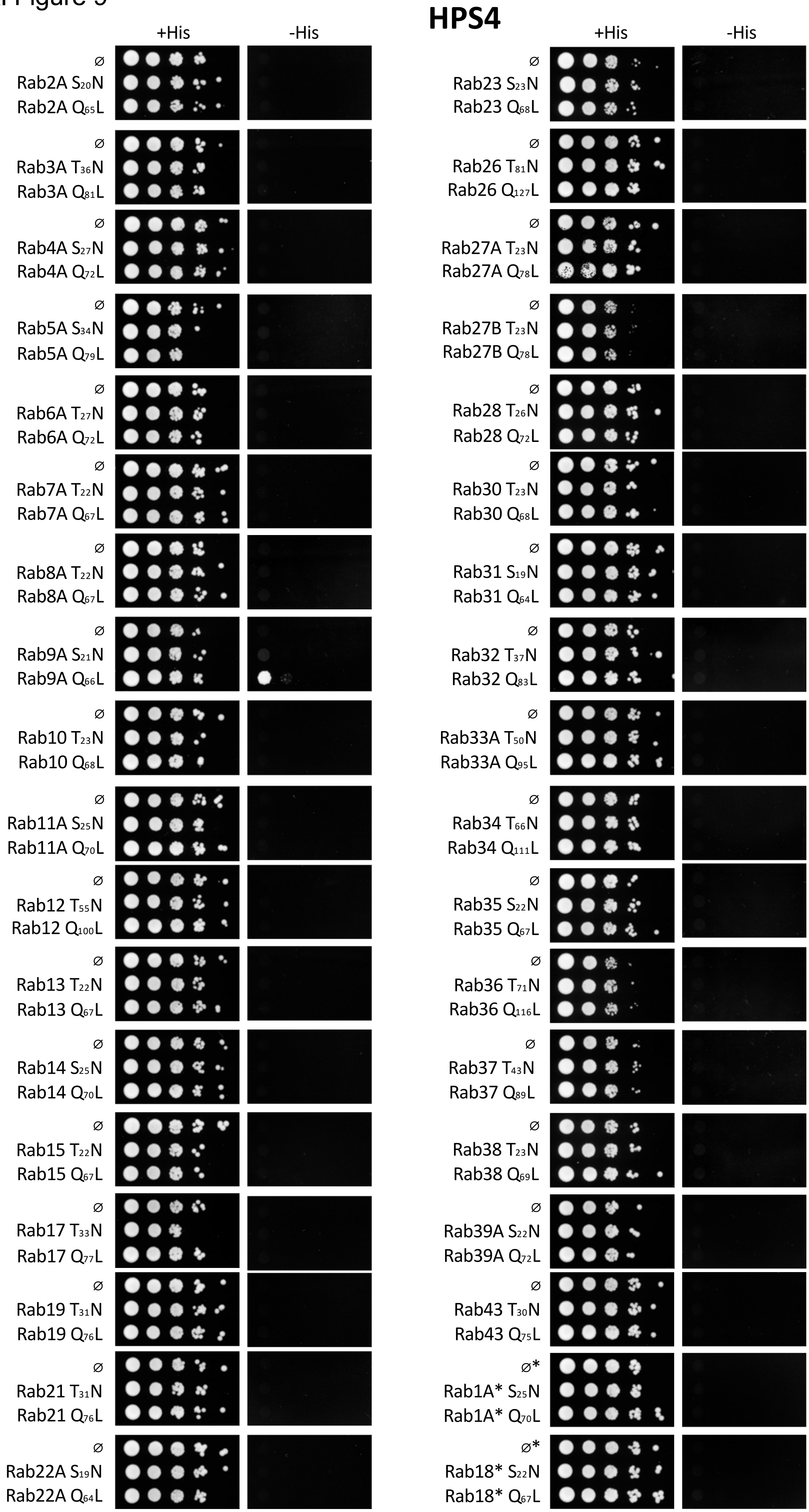

Supplemental Figure 10

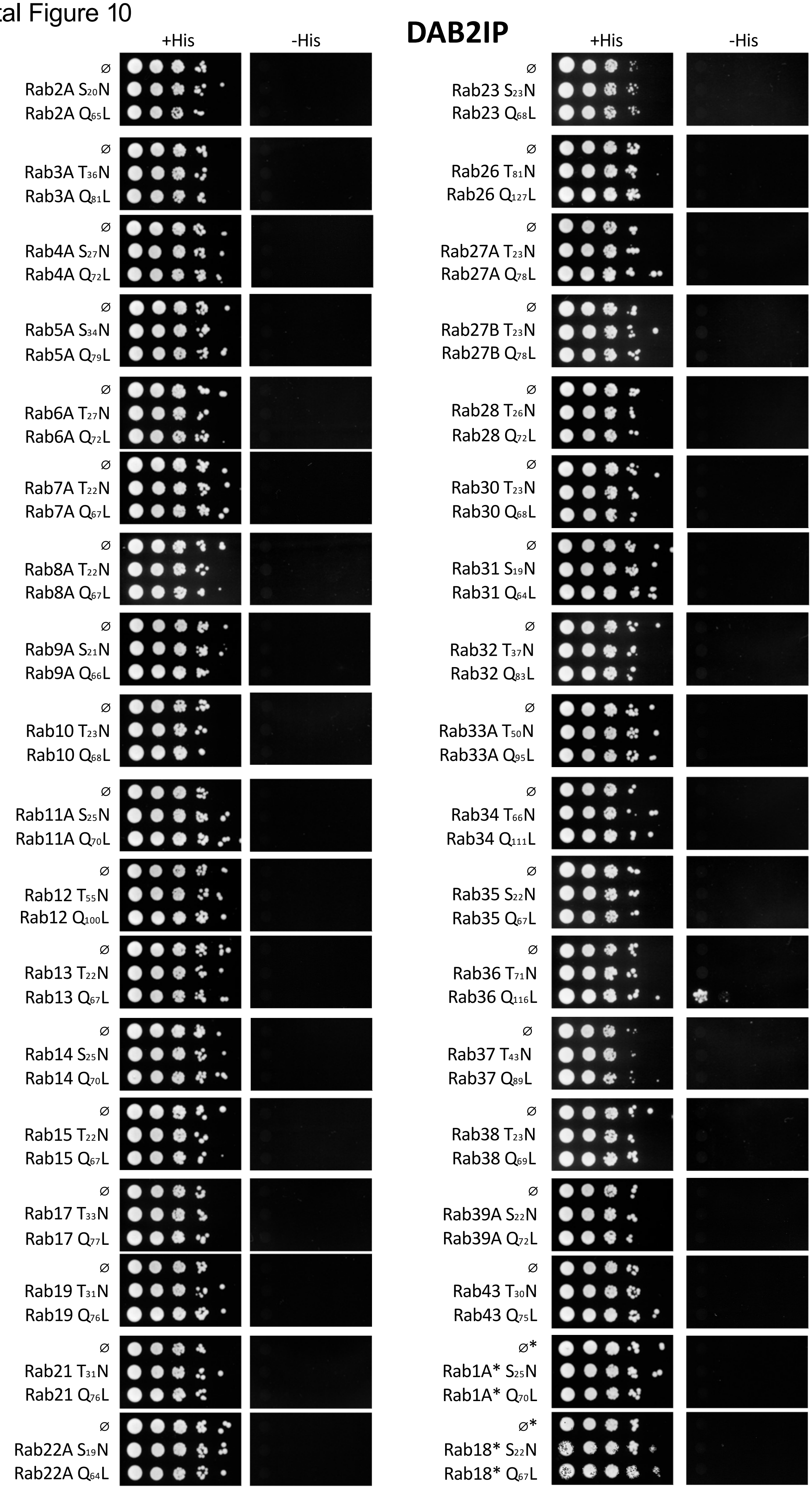

Supplemental Figure 11

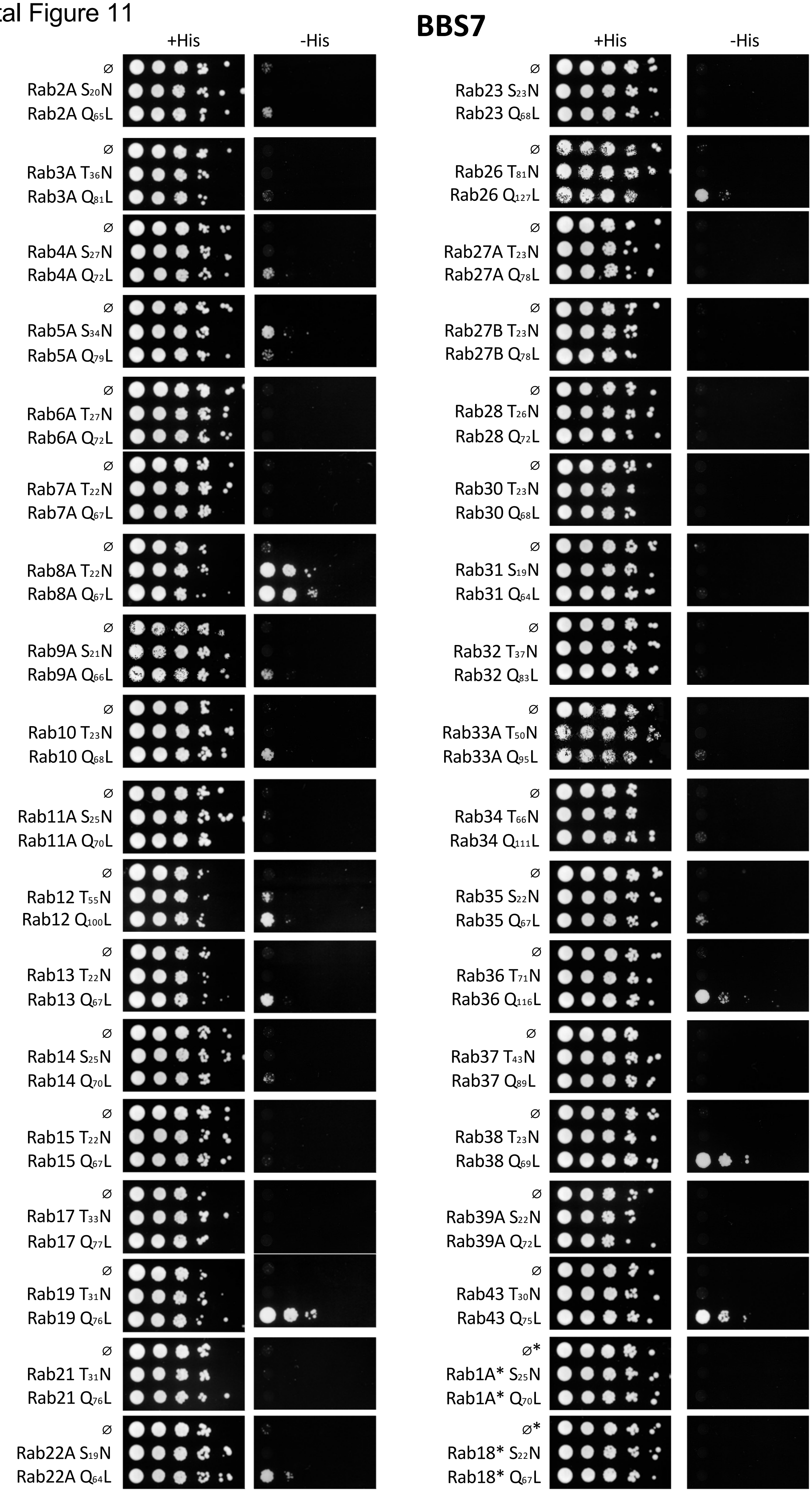

Supplemental Figure 12

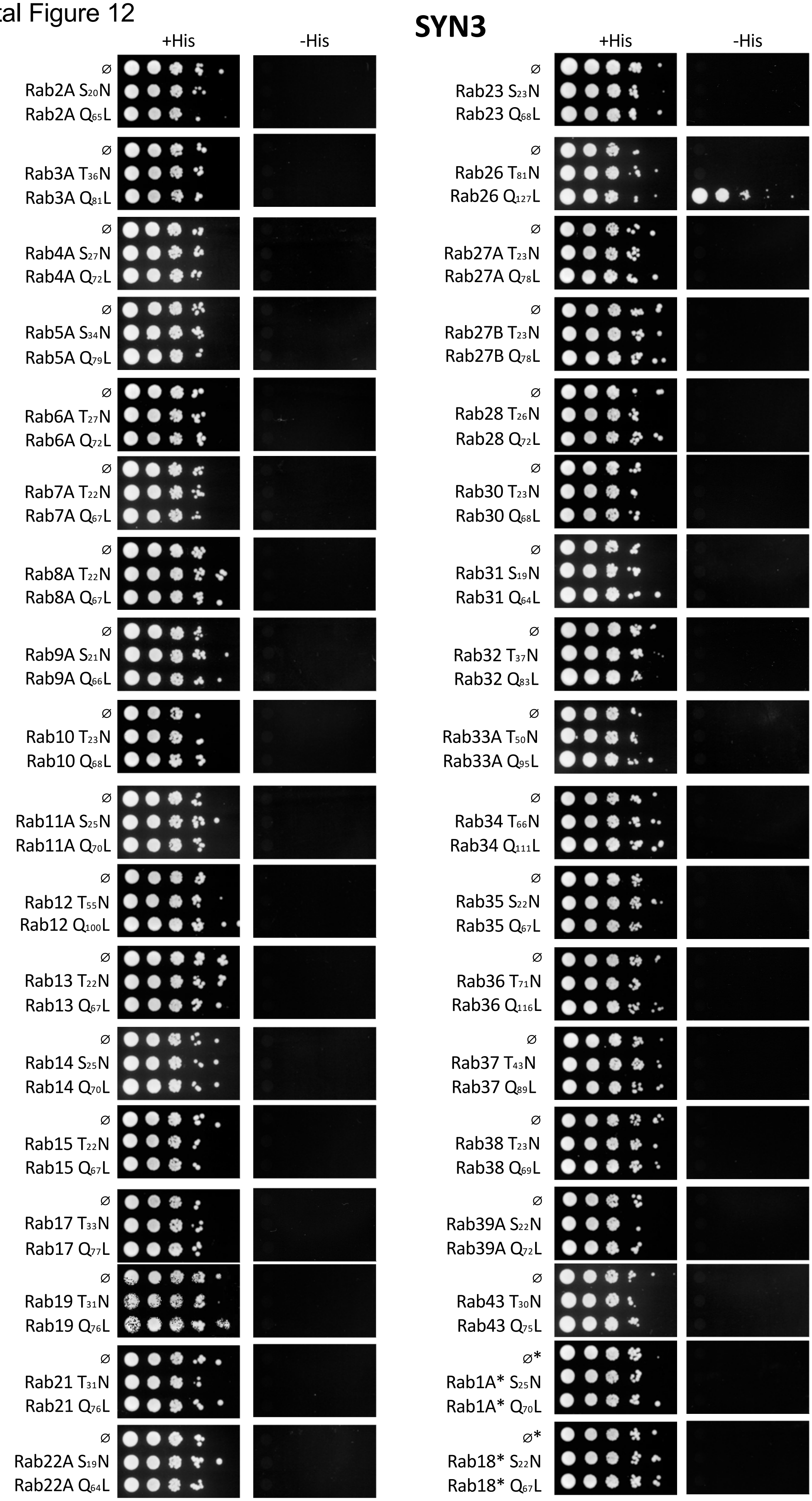

Supplemental Figure 13

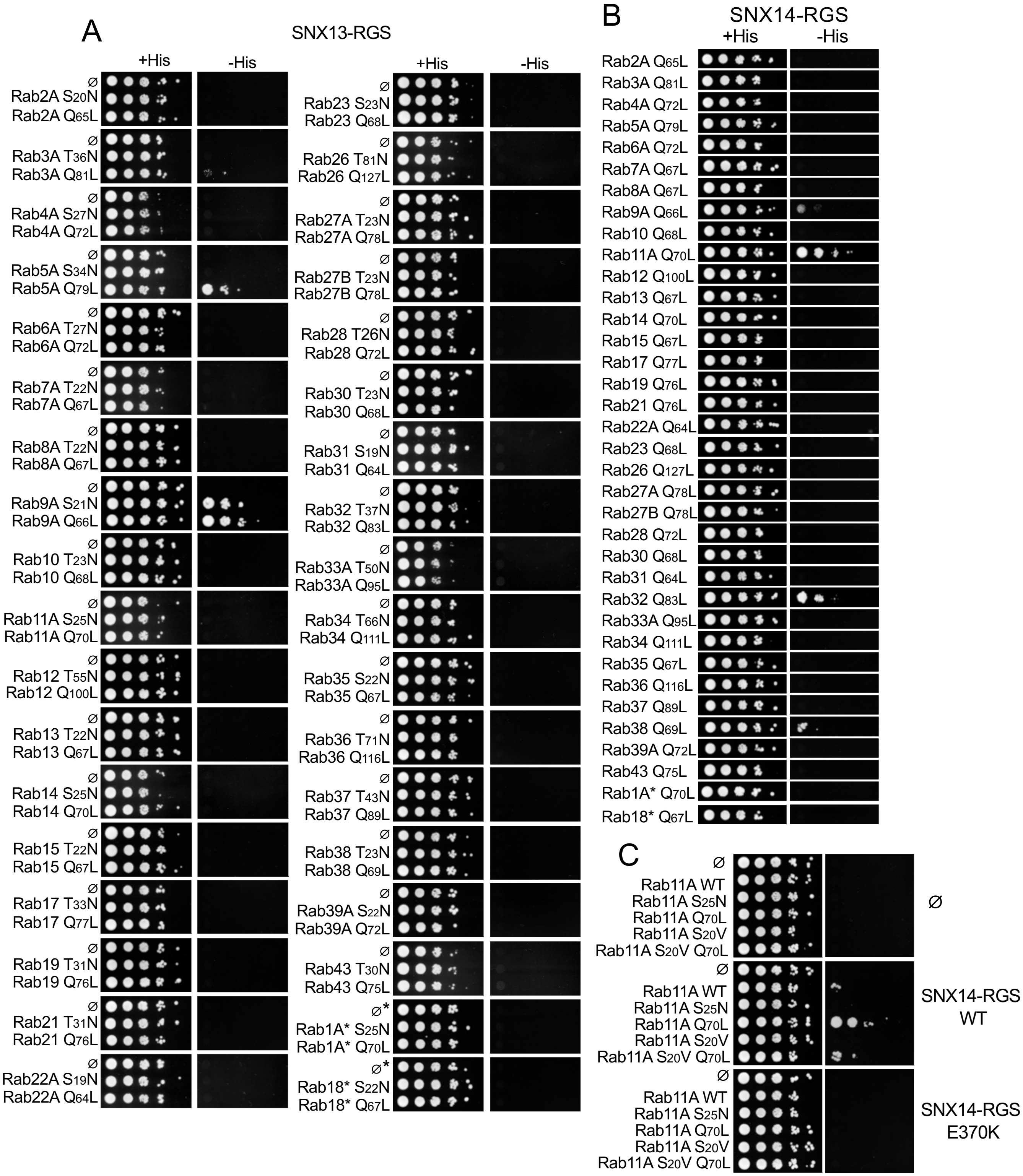

Supplemental Figure 13

D

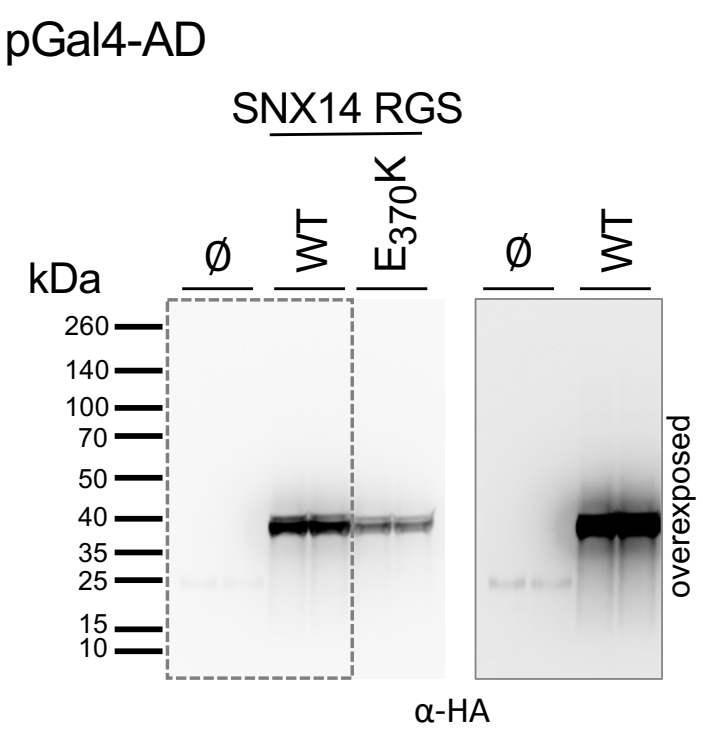

E

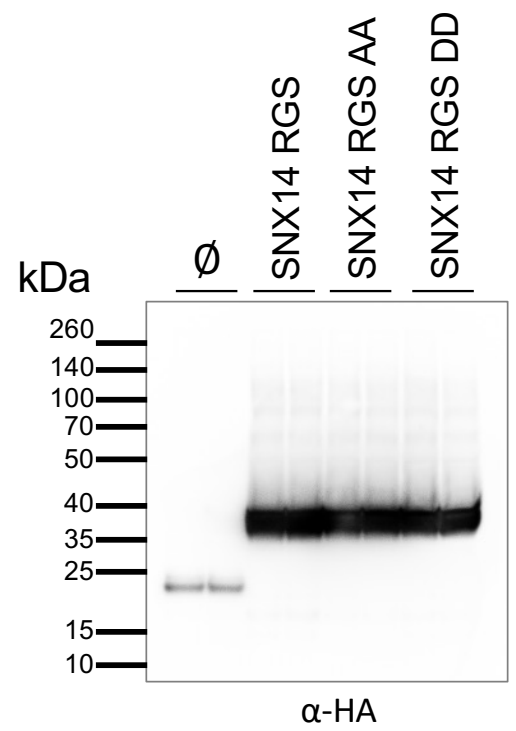

F

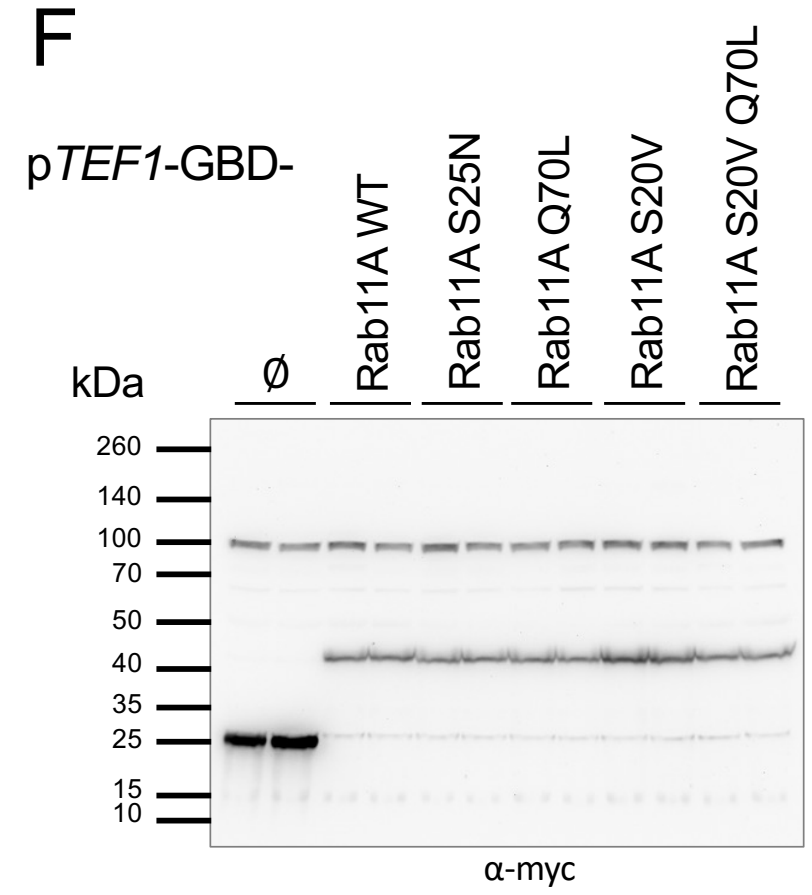

G

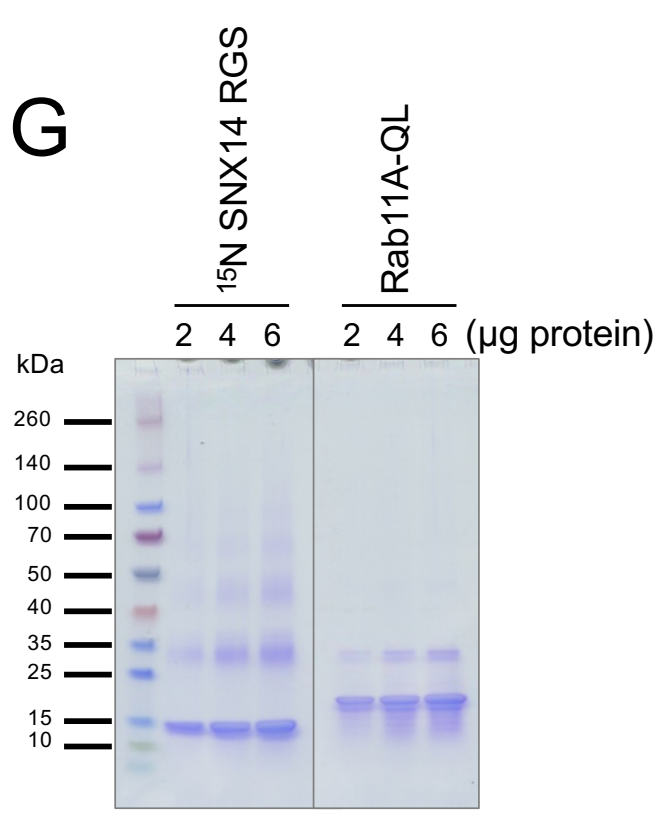

Supplement: Supplementary file 1 [file NIHPP2026.09.15.751902v2-supplement-1.pdf]
