# Supplementary material for: Survey of nucleotide-specific Rab GTPase interactions reveals multiple Rab effectors
Source: bioRxiv. 2026 Sep 17:2026.09.15.751902. Preprint. [Version 1] doi: 10.64898/2026.09.15.751902 (PMC13596360; doi:10.64898/2026.09.15.751902)
Supplement: Supplementary file 1 [file NIHPP2026.09.15.751902v1-supplement-3.pdf]

bioRxiv preprint doi: https://doi.org/10.64898/2026.09.15.751902; this version posted September 17, 2026. The copyright holder for this preprint (which was not certified by peer review) is the author/funder, who has granted bioRxiv a license to display the preprint in perpetuity. It is made available under a CC-BY 4.0 International license.

Supplemental Figure 1. Absence of self-activation by Rab GTPase baits.
Binary Y2H spot assays of each Rab GTPase bait (GTP-locked Q>L or GDP-locked S/T>N) paired with empty Gal4 activation domain vector, spotted onto permissive (+His) and selective (−His) plates. Absence of growth on −His confirms that no bait construct self-activates the reporter. Asterisks (*) denote Rab1A and Rab18, expressed from the reduced-strength Tef1* promoter.

Supplemental Figure 2. Full binary Y2H panel for MICAL-L1 and OPTN.
Binary Y2H spot assays for MICAL-L1 C-terminal fragment (residues 523–838, bMERB-containing) and OPTN N-terminal fragment (residues 45–339) against the complete Rab GTPase panel in GTP-locked (Q,

bioRxiv preprint doi: https://doi.org/10.64898/2026.09.15.751902; this version posted September 17, 2026. The copyright holder for this preprint (which was not certified by peer review) is the author/funder, who has granted bioRxiv a license to display the preprint in perpetuity. It is made available under a CC-BY 4.0 International license.

Q>L) and GDP-locked (T, S/T>N) conformations on permissive (+His) and selective (−His) plates. Highlighted interactions (green) were not previously reported in BioGRID.

Supplemental Figure 3. Full binary Y2H panel for RABEP1.
Binary Y2H spot assays for RABEP1 fragment i (residues 62–261) and fragment ii (residues 222–393) against the complete Rab GTPase panel on permissive (+His) and selective (−His) plates, for both GTP-locked (Q>L) and GDP-locked (S/T>N) Rab baits. Fragment i shows GTP-dependent interaction with multiple Rabs; fragment ii shows selective interaction with Rab5A and weak interaction with Rab26. Highlighted interactions (green) are novel.

Supplemental Figure 4. A. Binary screens of the Rab panel with the SNX-RGS domain recovered from DEEPN screen. **B.** Binary screens of the Rab panel of the GTP bound conformation against the SNX14 RGS domain. **C.** Y2H interaction of the indicated Rab 11 mutants with vector alone, the SNX14 RGS domain, of the RGS domain with the E370K mutation. Both the S20V and the Q70L mutations are thought to lock Rab11 in a GTP conformation. However, the S20V mutation on its own blocks binding of Rab11 to SNX14 RGS domain because it diminishes binding to the Q70L mutant when in combination. **D.** Levels of the wildtype and E370K mutant SNX14 RGS Gal4 activation domain fusion protein bait in duplicate transformants.

Supplemental Figure 1

Absence of self-activation by Rab GTPase 'baits'

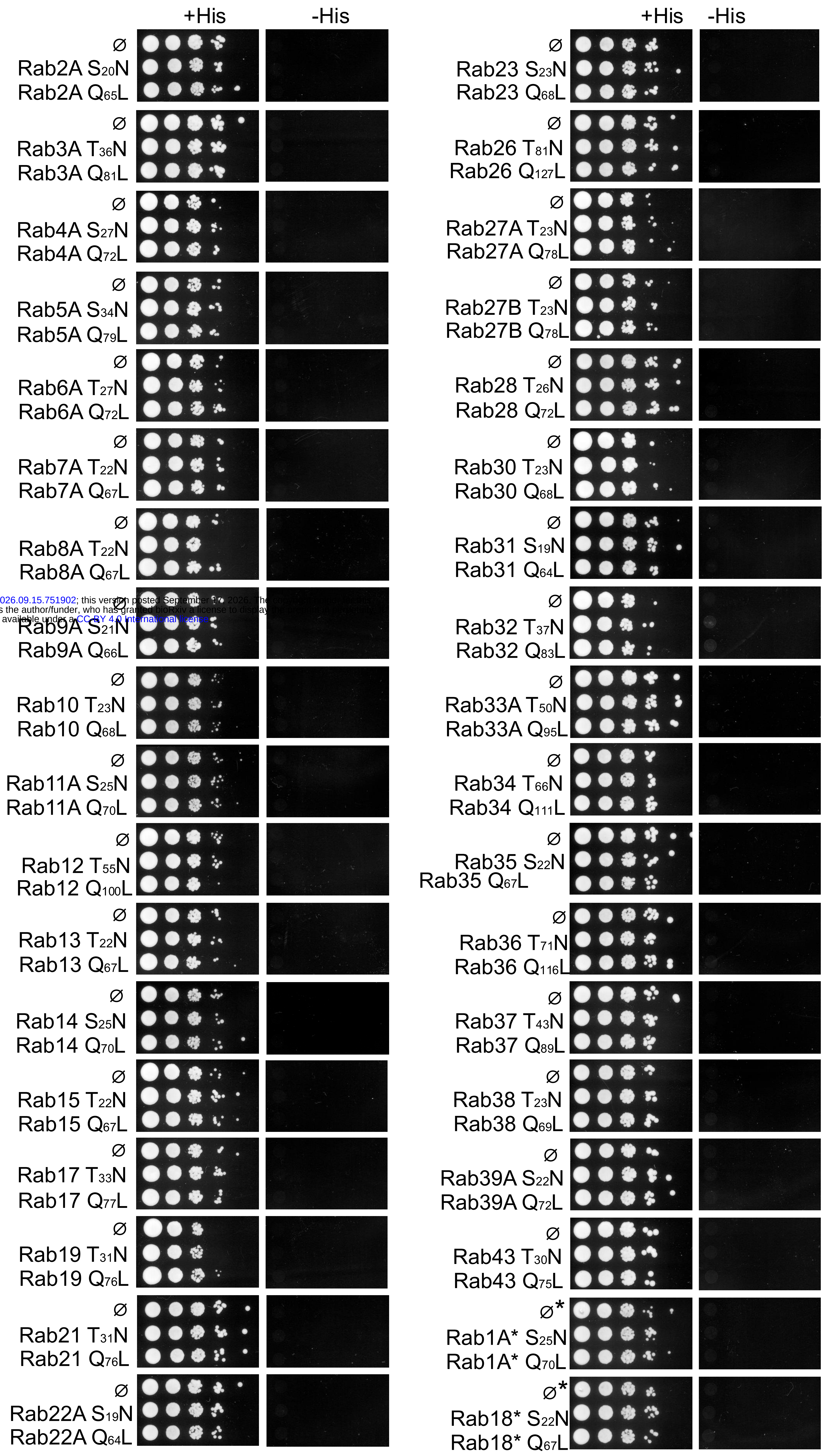

bioRxiv preprint doi: https://doi.org/10.64898/2026.09.15.751902; this version posted September 17, 2026. The [illegible]
preprint (which was not certified by peer review) is the author/funder, who has granted bioRxiv a license to display [illegible]
is made available under a CC-BY 4.0 International license.

Supplemental Figure 2

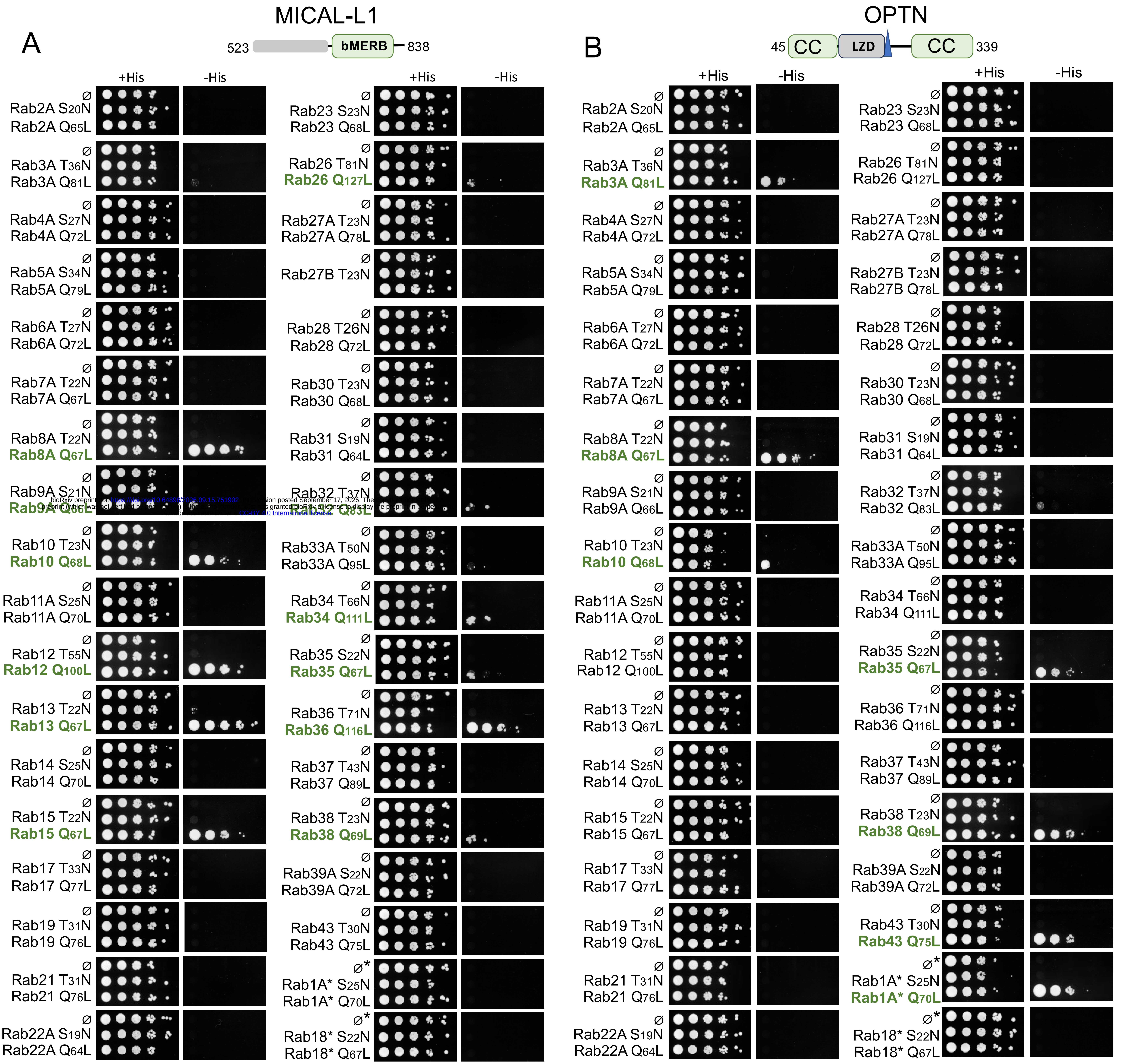

bioRxiv preprint doi: https://doi.org/10.64898/2026.09.15.751902; this version posted September 17, 2026. The copyright holder for this preprint (which was not certified by peer review) is the author/funder, who has granted bioRxiv a license to display the preprint in perpetuity. It is made available under a CC-BY 4.0 International license.

Supplemental Figure 3

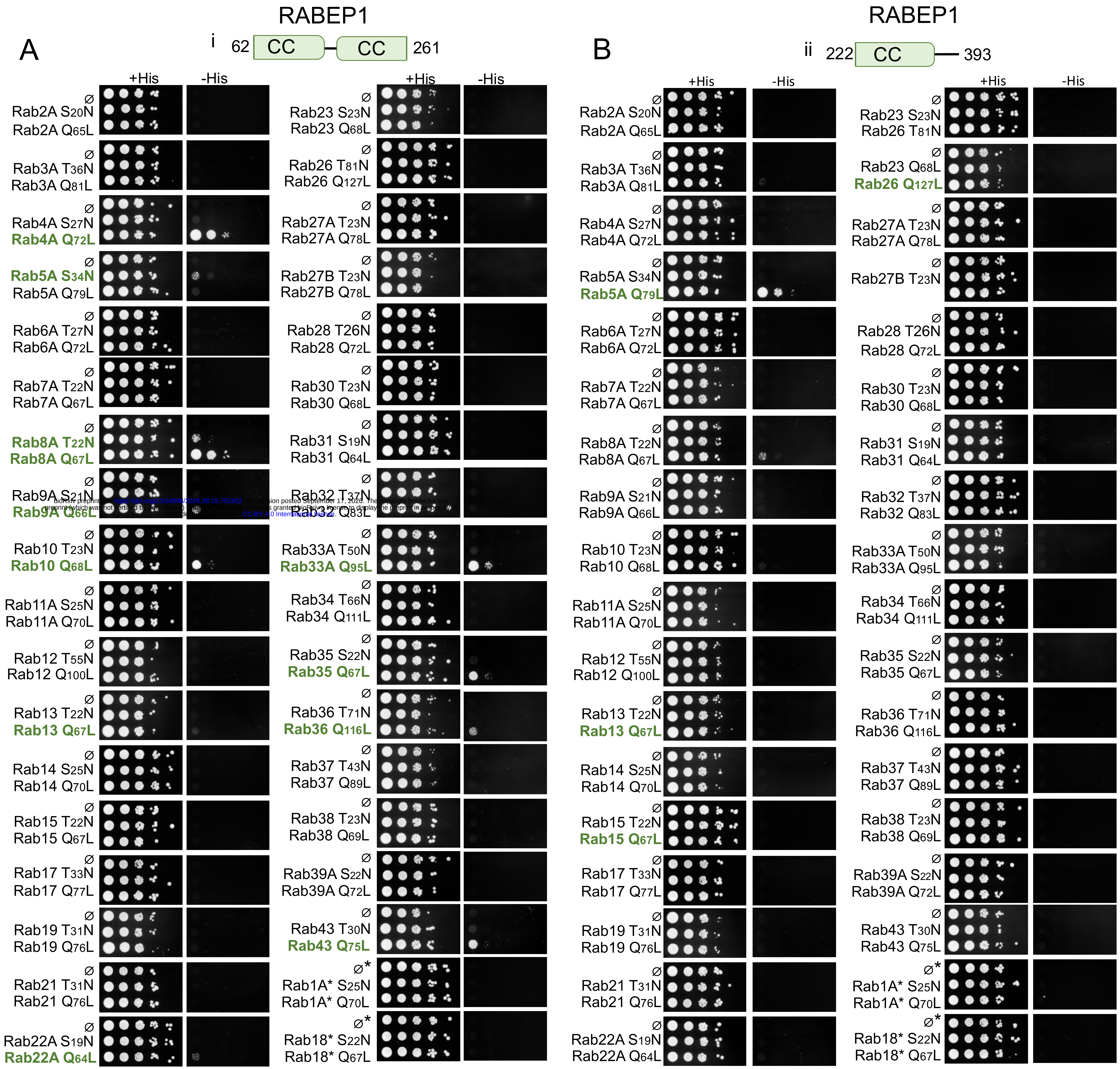

Supplemental Figure 4

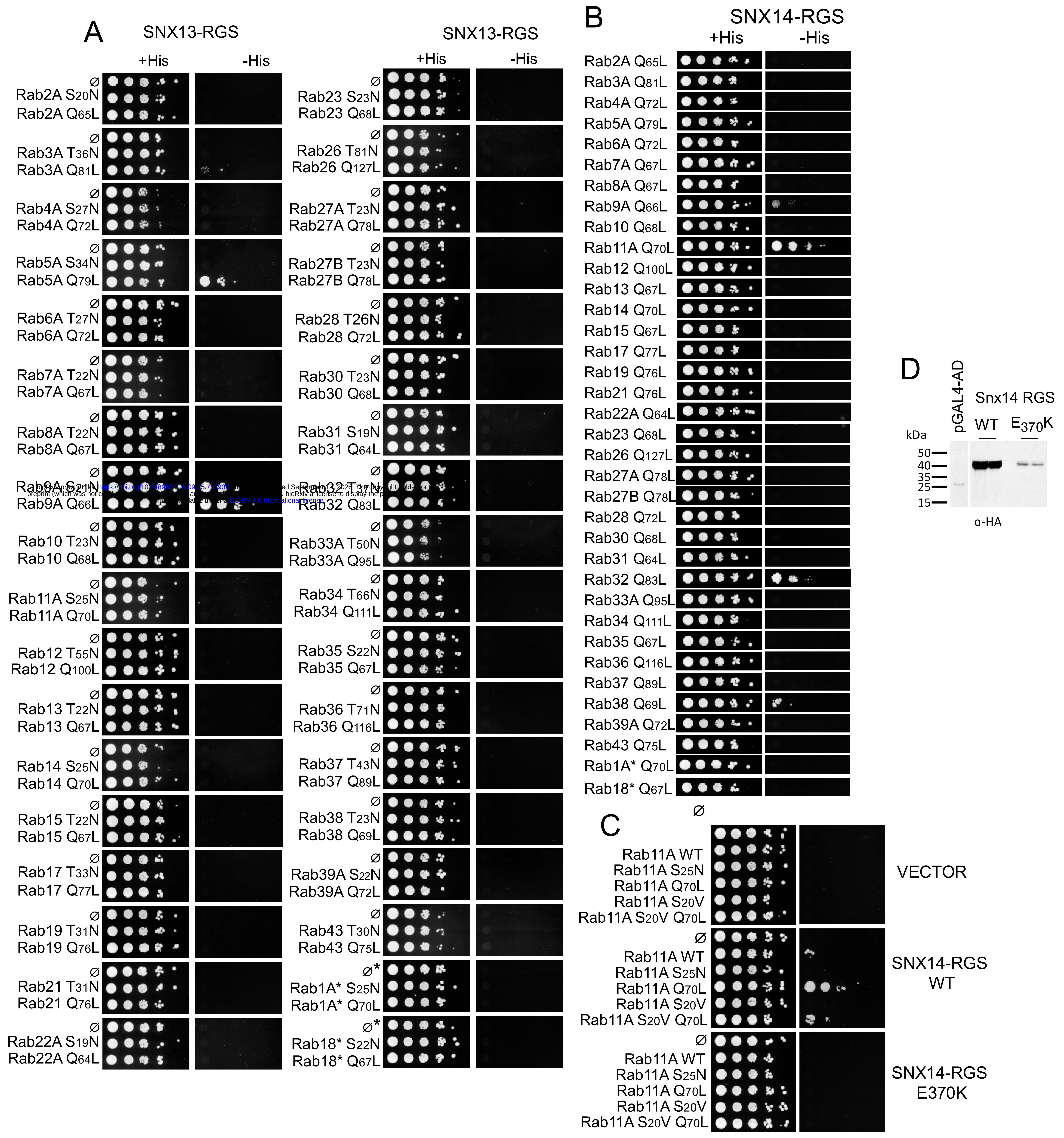

bioRxiv preprint doi: https://doi.org/10.64898/2026.09.15.751902; [illegible] September 17, 2026. The copyright holder for this preprint (which was not [illegible] bioRxiv a license to display the [illegible] made available under a CC-BY 4.0 International license.
